# Akt signaling is activated by TGFβ2 and impacts tenogenic induction of mesenchymal stem cells

**DOI:** 10.1186/s13287-021-02167-2

**Published:** 2021-01-26

**Authors:** Sophia K. Theodossiou, Jett B. Murray, LeeAnn A. Hold, Jeff M. Courtright, Anne M. Carper, Nathan R. Schiele

**Affiliations:** Chemical and Biological Engineering, University of Idaho, 875 Perimeter Dr. MS 0904, ID 83844 Moscow, USA

**Keywords:** Tendon, Tenogenesis, Mesenchymal stem cells, Tissue engineering, Akt, Smad3, TGFβ2

## Abstract

**Background:**

Tissue engineered and regenerative approaches for treating tendon injuries are challenged by the limited information on the cellular signaling pathways driving tenogenic differentiation of stem cells. Members of the transforming growth factor (TGF) β family, particularly TGFβ2, play a role in tenogenesis, which may proceed via Smad-mediated signaling. However, recent evidence suggests some aspects of tenogenesis may be independent of Smad signaling, and other pathways potentially involved in tenogenesis are understudied. Here, we examined the role of Akt/mTORC1/P70S6K signaling in early TGFβ2-induced tenogenesis of mesenchymal stem cells (MSCs) and evaluated TGFβ2-induced tenogenic differentiation when Smad3 is inhibited.

**Methods:**

Mouse MSCs were treated with TGFβ2 to induce tenogenesis, and Akt or Smad3 signaling was chemically inhibited using the Akt inhibitor, MK-2206, or the Smad3 inhibitor, SIS3. Effects of TGFβ2 alone and in combination with these inhibitors on the activation of Akt signaling and its downstream targets mTOR and P70S6K were quantified using western blot analysis, and cell morphology was assessed using confocal microscopy. Levels of the tendon marker protein, tenomodulin, were also assessed.

**Results:**

TGFβ2 alone activated Akt signaling during early tenogenic induction. Chemically inhibiting Akt prevented increases in tenomodulin and attenuated tenogenic morphology of the MSCs in response to TGFβ2. Chemically inhibiting Smad3 did not prevent tenogenesis, but appeared to accelerate it. MSCs treated with both TGFβ2 and SIS3 produced significantly higher levels of tenomodulin at 7 days and morphology appeared tenogenic, with localized cell alignment and elongation. Finally, inhibiting Smad3 did not appear to impact Akt signaling, suggesting that Akt may allow TGFβ2-induced tenogenesis to proceed during disruption of Smad3 signaling.

**Conclusions:**

These findings show that Akt signaling plays a role in TGFβ2-induced tenogenesis and that tenogenesis of MSCs can be initiated by TGFβ2 during disruption of Smad3 signaling. These findings provide new insights into the signaling pathways that regulate tenogenic induction in stem cells.

**Supplementary Information:**

The online version contains supplementary material available at 10.1186/s13287-021-02167-2.

## Introduction

Tendons, the collagenous musculoskeletal tissues that connect muscle to bone to enable movement, are frequently injured and heal poorly, leading to long-term loss of function [1]. Tendon injuries are common in both the general population and athletes, and limited clinical treatment options combined with their increasing incidence and high costs in terms of both healthcare and productivity losses make tendinopathies a significant public health concern [1–3]. Mesenchymal stem cells (MSCs) are attractive for use in regenerative therapies to treat tendon injuries, as they are relatively easy to isolate and can differentiate into a variety of tissue lineages, including tendon [4–9]. However, the limited understanding of the cell signaling pathways involved in tenogenesis (differentiation toward the tendon lineage) is a challenge for tissue engineering and regenerative approaches.

Currently identified tenogenic pathways include those related to transforming growth factor beta (TGFβ) signaling [10]. TGFβ has three isoforms (TGFβ1, 2, and 3), which, in canonical signaling, appear to depend on small mothers against decapentaplegic (Smad) 2/3 signaling [11, 12]. Smads are intracellular signaling proteins that regulate several cellular processes including growth, differentiation, and proliferation [13]. Previously, we and others showed that TGFβ2 is a potent inducer of tenogenesis in murine MSCs in vitro [9, 11, 13–19]. MSCs supplemented with recombinant human TGFβ2 significantly increased production of the tendon marker proteins scleraxis and tenomodulin over 21 days (d) in culture and underwent extensive morphology changes indicative of a transition towards tendon-like cells [14]. TGFβ2 also effectively induced tenogenesis in mouse embryonic fibroblasts grown in an in vitro tendon model [13]. Interestingly, the same study showed that when Smad4, the downstream effector of Smad2 and 3, was knocked out in mouse embryonic fibroblasts via adenovirus/Cre-mediated deletion, proliferation was disrupted, but the cells still produced scleraxis, indicating early tenogenesis was able to proceed [13]. Conversely, chemical inhibition of Smad2/3 in mouse embryonic day (E)9.5 limb buds resulted in loss of scleraxis expression in the developing tendons [11]. In a different study, following Cre-mediated deletion of Smad2/3 at postnatal day (P)2 and 4, mutant mice did not appear to have an altered tendon phenotype when evaluated at P14 [20], suggesting loss of Smad2/3 at later developmental stages is not disruptive to tendon formation. Collectively, while canonical TGFβ2 signaling likely depends on Smad2/3, this recent evidence suggests that other cell signaling pathways may be active in tenogenesis that are independent of Smad.

Though pathways beyond TGFβ and Smad2/3 have yet to be extensively investigated in tenogenesis, the phosphatidylinositol 3 kinase (PI3K)/Akt/mTORC1/P70S6K pathway has been implicated in the TGFβ-mediated tendon response to injury [21]. Treatment of murine MSCs with TGFβ1, which induces a pro-inflammatory and pro-fibrogenic response in tendon [22], activated (phosphorylated) Akt [21]. Akt, a kinase with extensive downstream roles in cell survival, growth, protein synthesis, and apoptosis, is activated by the phospholipid membrane-bound PI3K in response to several extracellular cues, including various growth factors, and potentially TGFβ signaling [23]. Once phosphorylated, Akt can indirectly activate the mammalian target of rapamycin or mTORC1 complex. mTOR knockout mice display abnormalities in the gross anatomy of their Achilles, patellar, and tail tendons [21], suggesting mTOR is involved in tendon development and maintenance. mTOR also activates protein S6 kinase, or P70S6K, which mediates protein synthesis and cell growth [24]. As P70S6K is a downstream effector of Akt, it is possible that P70S6K may be activated during TGFβ2-induced tenogenesis. P70S6K has not been investigated in the context of tenogenic differentiation, though it may contribute to proliferation and migration of other fibroblastic cells, such as lung fibroblasts [25]. Overall, although components of the PI3K/Akt/mTORC1/P70S6K pathway have been implicated in tendon development, interactions between this pathway and the tenogenic isoform of TGFβ, TGFβ2, have not been investigated.

Existing data suggest activation of Akt by TGFβ signaling can take place independently of Smad2 and 3 [26], but Akt activation has not been investigated in response to TGFβ2 specifically. Prior studies have mainly explored exogenous TGFβ1 to enhance TGFβ signaling in experimental settings. However, TGFβ isoforms induce differentiation towards distinct musculoskeletal tissue lineages. TGFβ3 is known to be chondrogenic [10], while TGFβ1 may induce chondrogenesis [27] or fibrosis and inflammation [28], and TGFβ2 is tenogenic [13, 14, 29, 30]. Thus, there remains a need to assess interactions between TGFβ2 and Akt signaling, specifically for tenogenesis. Notably, there is conflicting evidence as to whether Akt signaling enhances or attenuates cellular responses to TGFβ [31, 32], further highlighting the need for additional studies. Though Akt signaling is a precursor to a multitude of cellular events, establishing its potential involvement in tenogenesis is beneficial due to the limited understanding of the signaling events preceding tenogenic differentiation. Non-canonical and Smad-independent interactions between TGFβ and other signaling pathways have been documented in various cell types, and summarized in a comprehensive review [24]. While TGFβ is known to interact with bone morphogenic protein (BMP), mitogen-activated protein kinase (MAPK), Wingless/Integrated (Wnt) [33], Hedgehog (Hh), and Notch signaling [24], potential crosstalk between TGFβ and the Akt/mTORC1/P70S6K pathway is of particular interest in the context of tenogenesis due to the availability of therapeutic agents that can target Akt/mTORC1/P70S6K [34].

Taken together, additional signaling pathways may be involved in early TGFβ2-induced tenogenesis of stem cells, and one such pathway may be PI3K/Akt/mTORC1/P70S6K. We hypothesized that Akt/mTORC1/P70S6K would be activated during TGFβ2-induced tenogenesis, independent of Smad3 signaling. To test this, we treated mouse MSCs with TGFβ2 to induce tenogenic differentiation, and chemically inhibited Akt and Smad3 signaling for up to 7 d. We examined cell morphology, assessed activation of Akt, mTOR, and P70S6K, and evaluated levels of the tendon marker, tenomodulin. Our results showed that TGFβ2 activated Akt, and inhibiting Akt prevented TGFβ2-induced tenogenesis. Inhibiting Smad3 appeared to accelerate TGFβ2-induced tenogenesis. The tenogenic involvement of pathways other than Smad3 provides additional targets for investigations in tendon development.

## Materials and methods

### Cell culture and tenogenic induction

Murine MSCs (C3H10T1/2, ATCC, Manassas, VA), a model MSC used in prior studies investigating tenogenesis and tendon injury [21, 29, 35], were cultured and supplemented with TGFβ2 to induce tenogenesis as previously described [14]. Briefly, cells were expanded in standard growth medium (Dulbecco’s Modified Eagle’s Medium (DMEM), 10% fetal bovine serum (FBS), and 1% Penicillin/Streptomycin) until 70% confluent and used between passages 5 and 13. MSCs were trypsinized and seeded into each well of a 24-well plate. Cells used for 15 minutes (min), 30 min, 1 hour (h), and 24 h timepoints were seeded at 25,000 cells/cm^2^. Cells for 3 and 7 d timepoints were seeded at 5000 cells/cm^2^. Cells were incubated for 24 h to allow for initial cell attachment, and then washed with warmed phosphate-buffered saline (PBS) (Gibco, Grand Island, NY). The medium was switched to low-serum medium (DMEM, 1% FBS, 1% Penicillin/Streptomycin) and allowed to equilibrate for 24 h. Cells were rinsed with warm PBS and cultured for 15 min, 30 min, 1 h, 24 h, 3 d, 7 d, or 14 d in low-serum medium with the corresponding amount of sterile water (vehicle controls) or low-serum medium supplemented with 50 ng/mL recombinant human TGFβ2 (PeproTech, Rocky Hill, NJ). The medium was changed every third day. Experiments were repeated a minimum of 3 times.

### Inhibition of Akt and Smad3

To inhibit Akt signaling, cells were seeded in 24-well plates and cultured for 15 min, 30 min, 60 min, 24 h, 3 d, and 7 d in low-serum medium with water and dimethyl sulfoxide (DMSO) (vehicle controls), and low-serum medium supplemented with 50 ng/mL recombinant human TGFβ2 (PeproTech), 500 nM of the Akt inhibitor MK-2206 [36] (MedChem Express, Monmouth Junction, NJ), or both (TGFβ2 + MK-2206). To inhibit Smad3 signaling, cells were cultured and seeded into 24-well plates as described above for 15 min, 30 min, 60 min, 24 h, 3 d, and 7 d in low-serum medium with water and DMSO (vehicle controls), and low-serum medium supplemented with 50 ng/mL recombinant human TGFβ2 (PeproTech), 2 μM of the Smad3 inhibitor SIS3 [11] (Tocris, Bristol, UK), or both (TGFβ2 + SIS3).

### Western blot analysis

Cells were collected for western blot (WB) analysis in RIPA cell lysis buffer and HALT protease inhibitor (Invitrogen, Carlsbad, CA). Sodium dodecyl sulfate (SDS) was added at a 1:1 ratio and samples were sonicated, heated to 100° C for 5 min, and loaded into Novex Wedgewell 4–20% Tris Glycine Mini Gels (Invitrogen). Lanes were loaded differentially to normalize total protein content. Cell lysate collected from each well of the 24-well plate was run in its own lane (2 to 3 wells of each condition were run, analyzed, and averaged per individual experiment). Samples probed for mTOR and phosphorylated (P)-mTOR required 4–12% Tris Glycine gels (Invitrogen) due to the large protein size. Following electrophoresis, gels were transferred to nitrocellulose membranes (Invitrogen), blocked in 5% milk in Tris-buffered saline (Boston Bioproducts, Ashland, MA) with 0.1% Tween20 (TBST) (Acros Organics, Morris Plains, NJ), and incubated overnight at 4° C on an orbital shaker with appropriate primary antibodies in 5% bovine serum albumin (BSA) in TBST. Primary antibodies raised in rabbit were purchased for P-Smad3, β-actin (Abcam, Cambridge MA), mTOR, P-mTOR, Akt, P-Akt, P70S6K, P-P70S6K, Smad2/3 (Cell Signaling Technologies, Danvers, MA), and tenomodulin (Tnmd) (Invitrogen) and used at concentrations of 1:1000 to 1:10,000. Phosphorylation indicates activation, and increases in levels of P-Akt, P-mTOR, P-P70S6K, and P-Smad3 were used as representations of increased activation. Blots were washed 3× for 5 min in TBST and incubated for 1 h at room temperature with goat anti-rabbit HRP-linked secondary antibody (Invitrogen). Blots were then washed in TBST, developed using ECL chemiluminescence reagents (Invitrogen), imaged using a Genesis Pi6x imager (Syngene, Frederick, MD), and analyzed via band densitometry in ImageJ (NIH, Bethesda, MD), with all intensities normalized to their respective β-actin bands.

### Fluorescence staining and confocal microscopy

To observe changes in cell morphology, cells were cultured and supplemented with TGFβ2, MK-2206 and SIS3, as described above, but on glass coverslips. At 24 h, 3 d, and 7 d, the medium was removed, cells were rinsed with PBS, and fixed in 10% formalin overnight at 4° C. Cells were washed with PBS, permeabilized with 0.1% Triton-X (Acros Organics), and stained with FITC-phalloidin and 4,6-Diamidino-2-phenylindole (DAPI) (Life Tech., Waltham, MA) to observe the actin cytoskeleton and cell nuclei, respectively. Coverslips were mounted on slides and imaged on a spinning-disk confocal microscope (Nikon/Andor, Melville, NY).

### Statistical analysis

Proteins were initially normalized to their respective β-actin bands. As not all timepoints and experiments could be run on the same gel, treatment groups were normalized to their respective controls at each timepoint. Phosphorylated protein content was then normalized to total Akt, P70S6K, mTOR, or Smad2/3 content, as previously described [37]. Each experimental run was averaged (minimum *n* = 3 independent runs, 2–3 technical replicates per run), and ratios were calculated from bands imaged on the same membrane. Resulting ratios were analyzed using 1-way analysis of variance (ANOVA) with Sidak’s multiple comparison test (Prism 8, GraphPad, La Jolla, CA). Significance was set at *p* < 0.05. Results are reported as mean ± standard deviation.

## Results

### Akt signaling is activated during early TGFβ2-induced tenogenesis

**Fig. 1:**
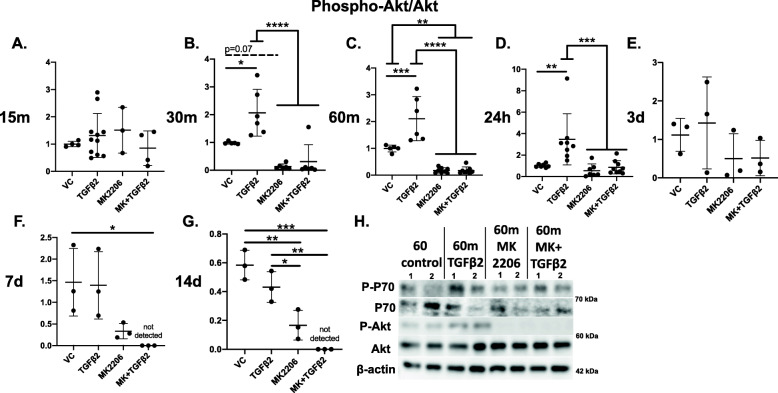
Akt is activated by TGFβ2 and inhibited with MK-2206 as a function of time. **a**–**g** Quantified western blot band densitometry showing the ratio of P-Akt to Akt as a function of time. The ratio of P-Akt to Akt was significantly increased by TGFβ2 treatment at **b** 30 m, **c** 60 m, and **d** 24 h. Akt activation levels in MSCs were significantly decreased by MK-2206 at **b** 30 m, **c** 60 m, **d** 24 h, **f** 7 d, and **g** 14 d. **h** Representative western blot showing MK-2206 prevents Akt activation at 60 m, while TGFβ2 increases Akt phosphorylation. P-P70S6K and P70S6K remained unaffected. *p < 0.05, **p < 0.01, ***p < 0.001, ****p < 0.0001. Bars = mean ± standard deviation

TGFβ2 treatment significantly increased the ratio of P-Akt (activated) to total Akt at 30 min (*p* < 0.05; Fig. 1b) and 60 min (*p* < 0.001; Fig. 1c) and at 24 h (*p* < 0.01; Fig. 1d), compared to controls. TGFβ2 increased the average P-Akt/Akt ratio, though not significantly, at 15 min and 3 d (Fig. 1a, e). Compared to controls, TGFβ2 did not alter the ratio of P-mTOR to mTOR at any timepoint (Fig. S1A-E). TGFβ2 significantly increased the ratio of P-P70S6K to P70S6K compared to controls at 24 h (*p* < 0.05; Fig. S2D), and P-P70S6K activation trended higher at 60 min (*p* = 0.08; Fig. S2C). TGFβ2 did not impact the ratio of P-P70S6K to P70S6K at 15 and 30 min or 3 and 7 d (Fig. S2A, B, E, F).

### Akt inhibition disrupts early TGFβ2-induced tenogenesis

**Fig. 2:**
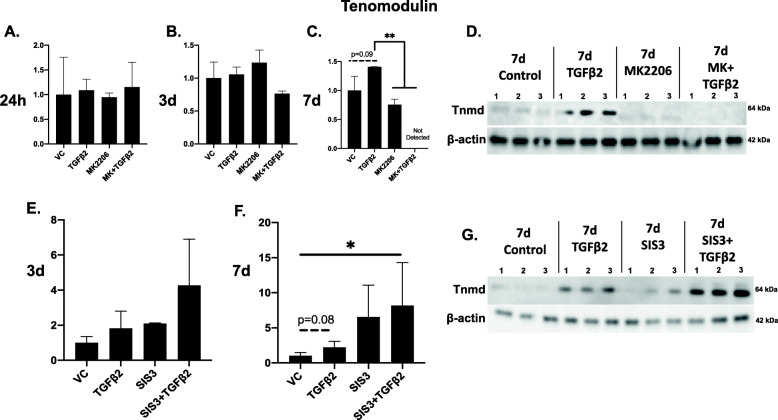
Akt inhibition prevents increases in tenomodulin, while Smad3 inhibition appears to accelerate tenomodulin production. **a**–**c** Quantified western blot band densitometry showing tenomodulin is not detectable following 7 d of treatment with MK-2206 + TGFβ2, indicating tenogenesis is disrupted when Akt signaling is inhibited. **d** Representative western blot showing cells treated with MK-2206 + TGFβ2 do not produce tenomodulin at 7 d. **e**, **f** MSCs treated with SIS3 + TGFβ2 produce more tenomodulin than cells treated with TGFβ2 alone at 7 d. **g** Representative western blot showing MSCs production of tenomodulin increases with 7 d of TGFβ2 and SIS3 treatment, compared to controls, and MSCs treated with TGFβ2 or SIS3 alone. *p < 0.05, **p < 0.01. Bars = mean ± standard deviation

**Fig. 3:**
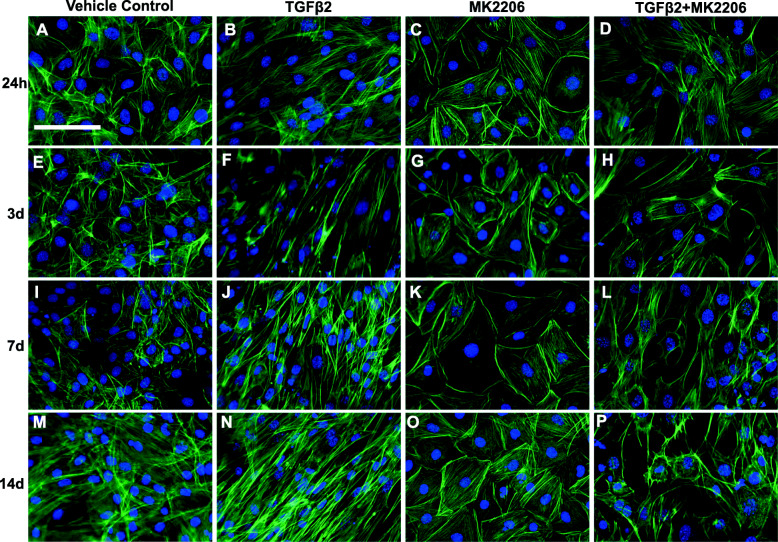
Akt inhibition impacts tenogenic cell morphology. Representative images (20×) of MSCs treated with TGFβ2 and MK-2206. Actin cytoskeleton (green) and cell nuclei (blue) are shown in MSCs at 24 h, and 3, 7, and 14 d (**a**–**p**). **d**, **h**, **l**, **p** MSCs treated with both TGFβ2 and the Akt inhibitor, MK-2206, had disruptions in cell morphology and did not appear elongated or fibroblastic, compared to **b**, **f**, **j**, **n** cells treated with TGFβ2 only and **a**, **e**, **i**, **m** controls. **c**, **g**, **k**, **o** MK-2206 alone did not appear to drastically impact cell morphology. Scale bar = 100 μm

Cells were treated with TGFβ2, MK-2206, or MK-2206 + TGFβ2. MK-2206 effectively decreased Akt activation both on its own and when used in combination with TGFβ2. The ratio of P-Akt to Akt was significantly lower in both MK-2206 and MK-2206 + TGFβ2 treated cells, compared to TGFβ2-only treated cells at 30 min (*p* < 0.0001; Fig. 1b), compared to all other groups at 60 min (*p* < 0.01 to *p* < 0.001; Fig. 1c, h), and compared to TGFβ2-only treated cells (*p* < 0.001) at 24 h (Fig. 1d). The P-Akt to Akt ratio remained lower in MK-2206-treated groups, and P-Akt was not detected in MK-2206 + TGFβ2 treated cells at 7 d (Fig. 1f) and 14 d (Fig. 1g). MK-2206 did not significantly impact the ratio of P-mTOR to mTOR at most timepoints (Fig. S1). At 24 h, mTOR activation was significantly lower in MK-2206-only treated cells, compared to TGFβ2-only cells (*p* < 0.05; Fig. S1D), and at 3 d, activation was significantly lower in MK-2206 + TGFβ2 treated cells, compared to TGFβ2-only cells (*p* < 0.05; Fig. S1E). MK-2206 did not affect the ratio of P-P70S6K to P70S6K at most timepoints, though P-P70S6K activation trended lower (*p* = 0.08) in MK-2206 and MK-2206 + TGFβ2 treated cells at 60 min (Fig. S2C) and was significantly lower (*p* < 0.05) in MK-2206 treated cells compared to TGFβ2-only treated cells at 24 h (Fig. S2D). At this same timepoint, MK-2206 + TGFβ2 treated cells had a significantly higher ratio of P-P70S6K to P70S6K compared to vehicle controls (*p* < 0.05; Fig. S2D), and the ratio in MK-2206 + TGFβ2 treated cells trended higher (*p* = 0.055) than in MK-2206-only treated cells. At 7 d, levels of tenomodulin were undetectable in the MK-2206 + TGFβ2-treated cells, indicating tenogenesis had been disrupted (*p* < 0.01; Fig. 2c, d). Tenogenic cell morphology was disrupted with MK-2206 + TGFβ2 treatment. Cells showed reduced elongation, proliferation, and localized alignment, compared to cells treated with TGFβ2 alone, at 24 h, 3 d, and 7 d (Fig. 3). Finally, although cells treated with MK-2206 + TGFβ2 had a significantly higher ratio of P-Smad3 to Smad2/3 at 60 min (*p* < 0.05; Fig. S3A), levels of activated Smad3 were similar to controls by 24 h (Fig. S3B).

### Early TGFβ2-induced tenogenesis proceeds when Smad3 signaling is disrupted

**Fig. 4:**
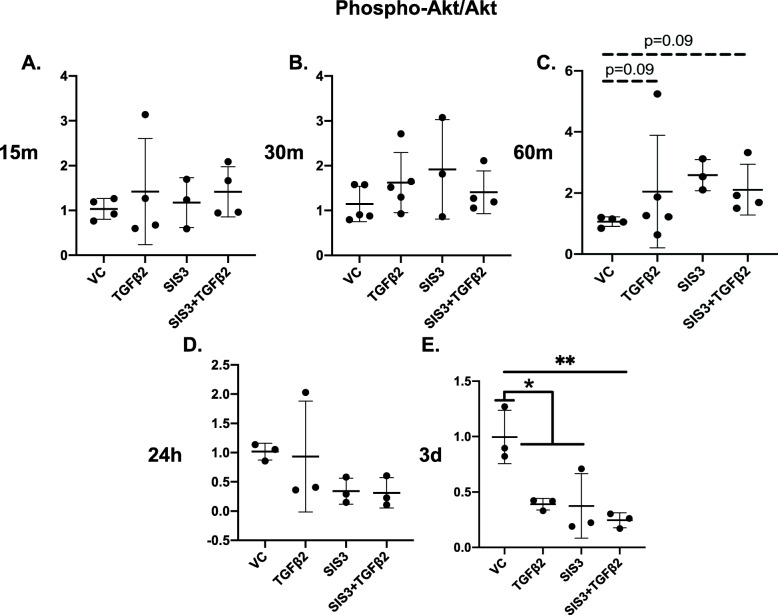
Smad3 inhibition has limited impact on Akt activation. Quantified western blot band densitometry showing the ratio of P-Akt to Akt in MSCs treated with SIS3 to block Smad3 signaling. The ratio of P-Akt to Akt was not significantly altered by SIS3 at **a** 15 m, **b** 30 m, and **d** 24 h. **c** Akt activation trended higher in cells treated with TGFβ2 and SIS3 + TGFβ2 at 60 m (*p* = 0.09). **e** Akt activation levels were significantly decreased in all conditions except vehicle controls by 3 d. *p < 0.05, **p < 0.01. Bars = mean ± standard deviation

**Fig. 5:**
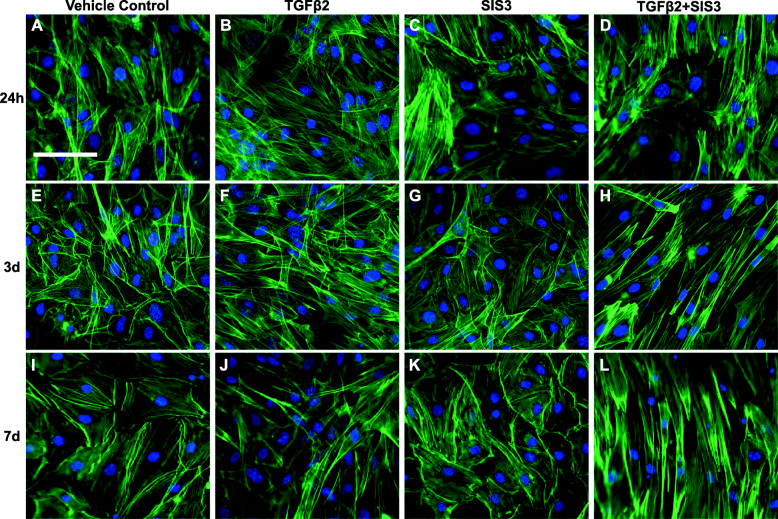
Smad3 inhibition does not alter tenogenic cell morphology. Representative images (20×) of TGFβ2 and SIS3-treated MSCs. **a**–**h** Actin cytoskeleton (green) and cell nuclei (blue) are shown in MSCs at 24 h, 3 d, and 7 d. **d**, **h**, **l** MSCs treated with both TGFβ2 and the Smad3 inhibitor, SIS3, appeared more fibroblastic and elongated, compared to **a**, **e**, **i** controls and **b**, **f**, **j** cells treated with TGFβ2 only. **c**, **g**, **k** SIS3 alone did not appear to impact cell morphology. Scale bar = 100 μm

Cells were treated with TGFβ2, SIS3, or SIS3 + TGFβ2. Cells treated with SIS3 had decreased activation of Smad3 and a lower ratio (*p* = 0.08) of P-Smad3 to total Smad2/3 at 60 min, as expected (Fig. S3C). SIS3 did not decrease the ratio of P-Akt to Akt alone or in combination with TGFβ2 at any timepoint (Fig. 4), though all conditions had significantly lower Akt activation compared to controls at 3 d (Fig. 4e). SIS3 did not impact the ratio of P-mTOR to mTOR, except at 15 min, where SIS3 + TGFβ2 treated cells had significantly lower mTOR activation compared to other timepoints (*p* < 0.01; Fig. S4A). Finally, at 15 min, SIS3 and SIS3 + TGFβ2 treated cells had a significantly lower ratio of P-P70S6K to P70S6K (*p* < 0.05; Fig. S5A), compared to cells treated with TGFβ2 alone, but SIS3 did not affect this ratio at any other timepoint (Fig. S5). Inhibiting Smad3 signaling with SIS3 did not prevent TGFβ2-induced tenogenesis. The morphology of cells treated with SIS3 + TGFβ2 displayed changes consistent with tenogenesis at earlier timepoints, compared to cells treated with TGFβ2 alone (Fig. 5). Levels of tenomodulin were also significantly higher (*p* < 0.01) at 7 d in cells treated with SIS3 + TGFβ2, compared to the TGFβ2-only group (Fig. 2e, f, g).

## Discussion

In this study, we showed that TGFβ2 activated Akt signaling during the initiation of tenogenesis, that inhibiting Akt signaling disrupted early TGFβ2-induced tenogenesis and that TGFβ2-induced tenogenesis proceeded despite disruption of Smad3. Activation of mTOR and P70S6K, which are downstream in the Akt signaling pathway, also changed in response to TGFβ2, though these changes appeared muted compared to the impact of TGFβ2 on Akt activation. Inhibiting Akt and Smad3 had limited impact on the activation of these downstream effectors (mTOR and P70S6K). Finally, we showed that disrupting Smad3 signaling during TGFβ2-induced tenogenesis appeared to accelerate morphological changes and tenomodulin production by MSCs. While previous work has shown that TGFβ signaling can proceed independently of Smad functionality [13], to our knowledge, this is the first study to investigate the activation of potential alternative cellular signaling pathways during early TGFβ2-induced tenogenesis.

Our results suggest that Akt activation is a possible tenogenic pathway that may regulate the cellular response to TGFβ2-induced tenogenesis. TGFβ2-treatment alone significantly increased Akt activation early in tenogenic induction (Fig. 1). Additionally, Akt activation with TGFβ2 across various timepoints showed an increasing trend up until 24 h, and a decreasing trend until 14 d, where the P-Akt to Akt ratio is significantly lower compared to 24 h (Fig. S6). Although the ratio of P-Akt to Akt increased by a maximum of only ~ 2.5:1 in response to TGFβ2, as a kinase, Akt activates a cascade of intracellular events, and only modest increases in signaling may be needed to induce robust changes in transcription and protein synthesis [23]. While MK-2206 prevented Akt activation by TGFβ2 and diminished tenogenic markers, levels of total (non-phosphorylated) Akt, as well as levels of phosphorylated and non-phosphorylated mTOR and P70S6K, were not changed by MK-2206 addition alone, except at some later timepoints. This suggests that even low levels of P-Akt may be enough to maintain some downstream signaling, that other non-canonical pathways may be maintaining mTOR and P70S6K signaling in the absence of Akt activation, or that these downstream signaling molecules are not involved in TGFβ2-induced tenogenesis. Overall, our findings show that TGFβ2 activates Akt signaling during early tenogenic induction.

Interestingly, in addition to not decreasing in response to MK-2206, mTOR activation was not significantly altered by TGFβ2 treatment and the apparent changes in Akt activation. This finding could be explained by Akt only indirectly activating mTOR in the canonical PI3K/Akt/mTORC1/P70S6K pathway, and this activation may be tissue-specific [23, 38]. The TGFβ-induced epithelial-to-mesenchymal transition is also regulated by mTOR [39]. Since TGFβ2-induced tenogenesis in these MSCs is occurring after the epithelial-to-mesenchymal transition that occurred in utero prior to MSC isolation, mTOR regulation may not directly influence tenogenic differentiation. It is also possible that mTOR activation is exclusive to tendon injury [21] and not activated during tenogenic differentiation. However, mTOR knockout mice display some abnormalities in gross tendon morphology [21], suggesting mTOR is involved in tendon development. Similarly, the ratio of P-P70S6K to P70S6K was not significantly increased at early timepoints. However, unlike mTOR, P70S6K activation was significantly higher at 24 h with TGFβ2 treatment. The significant increases in P70S6K activation at later timepoints (24 h) suggest some mTOR activation, and the delayed peak in activation is logical given its position downstream in the pathway. Ultimately, both mTOR and P70S6K showed some increases (though not significant) in activation in response to TGFβ2 at earlier timepoints, indicating that the PI3K/Akt/mTORC1/P70S6K pathway remains a potential driver of TGFβ2-induced tenogenesis.

Akt may allow TGFβ2-induced tenogenesis to proceed despite disruption of Smad3. While Smad3-independent activation of Akt by TGFβ signaling is not a novel finding [12, 26], TGFβ2-induced tenogenesis during disruption of Smad3 has not been previously shown. Inhibiting Smad3 with SIS3 did not decrease activation of Akt (Fig. 4) or downstream effectors, mTOR (Fig. S4, S5), except at isolated, later timepoints. While Akt activation did not increase significantly in the SIS3 + TGFβ2 treated cells, levels of P-Akt trended higher (*p* = 0.09) at 60 m (Fig. 4c), and it is possible that low levels of Akt activation are sufficient for tenogenesis to proceed. Unlike Akt, P-P70S6K activation showed an early, transient significant decrease in SIS3-treated cells at 15 min, but the ratio of P-P70S6K to P70S6K was otherwise similar to other groups at all timepoints (Fig. S5). The cause of this pattern is not clear, but could be due to effects of endogenous TGFβ1 on P70S6K signaling being disrupted by SIS3 [38]. As the three isoforms of TGFβ have partially overlapping but distinct roles in musculoskeletal tissue development [40], future studies are needed to determine the specific downstream effects of each TGFβ isoform within the context of Akt signaling. Furthermore, while other pathways not assessed in this study may be initiating tenogenesis in response to TGFβ2 addition, Akt is a promising candidate due to the extensive morphological changes observed with Akt inhibition (Fig. 3). Akt inhibition does not lead to alterations in cell morphology, except in the MK-2206 + TGFβ2 groups. Similarly, SIS3 alone appears to do little to cell morphology or protein production, until combined with TGFβ2. While qualitative, the images of cell morphology showed enhanced tenogenic morphology with Smad3 inhibition and extensive disruption of tenogenic morphology with Akt inhibition. The novel finding of disrupted tenogenic morphology with Akt inhibition, along with loss of tenomodulin production, suggests that the Akt pathway is playing a role in early TGFβ2-induced tenogenesis.

The finding that TGFβ2 not only induces tenogenesis when Smad3 is disrupted, but that differentiation may be accelerated, is consistent with recent evidence that the downstream effector of Smad3 signaling (e.g., Smad4) may not be necessary for TGFβ2-induced tenogenic differentiation of mouse embryonic fibroblasts [13]. However, loss of Smad4 in tendon cells (via crossing Smad4 floxed mice with scleraxis-cre) resulted in joint contracture and a reduction in collagen fibril diameter, cellularity, and extracellular matrix volume in a murine model of Smad4 deletion by P5 and P14 [20]. The same study also showed that disrupting canonical TGFβ signaling using Smad2 and 3 mutant mice did not result in joint contracture when tamoxifen was administered on P2 and P4, but joint contracture was largely recapitulated by disrupting BMP signaling [20]. Taken together, our present work and these other recent studies suggest that Smad2 and 3 deletion does not disrupt TGFβ signaling in tenogenesis, but further research is needed to untangle the individual roles of Smad2 and 3, as well as the BMP-associated Smads1/5/8 [20].

Smad4 was also shown to play a role in cell proliferation in response to TGFβ2 [13]. While cellular proliferation was not quantified in this study, images show that changes in cell morphology (elongation and localized alignment) with SIS3 + TGFβ2 treatment are not accompanied by the large increases in cell number observed in prior studies of TGFβ2-only treated cells [14], suggesting Smad3 and hence downstream Smad4 signaling were effectively inhibited and may also impact proliferation. Finally, unlike TGFβ2-only and SIS3-only treated cells, most SIS3 + TGFβ2-treated groups did not survive past 10 d in culture, suggesting long-term SIS3 accumulation or disruption of Smad3 and downstream effectors is problematic for TGFβ2-induced tenogenesis. For this reason, only timepoints up until a maximum of 7 d are included in the data analysis. Overall, Smad4 appears to be necessary for maintenance of tenogenesis, but the role of Smad signaling in early tenogenesis and the dependence of TGFβ2-induced tenogenesis on Smad functionality are less clear.

It is possible that the apparent acceleration in TGFβ2-induced tenogenesis with Smad3 disruption is due to disruptions of cell signaling associated with endogenous TGFβ1. Fibroblasts and MSCs that are precursors to musculoskeletal tissues produce TGFβ1 during development [29, 30, 41–43], and TGFβ1 signaling is known to participate in the tendon response to injury and fibrosis [28, 44–46]. Multiple TGFβ isoforms may be active at any given time, but not all activate the same pathways, especially when non-canonical signaling is considered [47]. During early musculoskeletal tissue differentiation, TGFβ1 signaling may proceed exclusively via Smad, while TGFβ2 may be able to induce differentiation via alternate pathways that other TGFβ isoforms do not activate [12]. As TGFβ1 may not be tenogenic and in other studies is chondrogenic [48, 49] or fibrogenic [28], disrupting Smad3 may prevent the non-tenogenic effects of TGFβ1. Simultaneously, TGFβ2 signaling may proceed via an alternate pathway, thus accelerating tenogenesis when competing signals from endogenous TGFβ1 are disrupted. Other aspects of tendon development, such as production of matrix components like fibronectin and proteoglycans, as well as collagen production and organization, may require Smad4-dependent [20] and independent [50] TGFβ1 signaling, which long-term Smad3 inhibition prohibits. Furthermore, injury in a neonatal tendon is associated with Smad2/3 activation, and Smad2/3 activation meditated by TGFβ signaling through the TGFβ type I receptor ALK4/5/7 impacts regenerative healing [51]. Another Smad protein, Smad8, has also been shown to enhance regeneration of tendon injuries when MSCs are genetically engineered to overexpress both Smad8 and its downstream target, BMP2 [52, 53], suggesting that Smad signaling may be involved in tendon regeneration. Finally, interactions between multiple TGFβ isoforms and other Smads, such as Smad8 [52, 53], and downstream Smad-dependent signals, including BMPs [20, 24, 54–56], may be necessary for continued or postnatal tendon differentiation and maintenance. Interactions between Smad signaling and all three TGFβ isoforms, as well as interactions with BMP signaling, will be explored in future long-term studies. Taken together, Smad activation by TGFβ family members may play unique and time-dependent roles in differentiation and regeneration, but more work is needed to elucidate the impact of Smads in regulating tendon formation.

This study is not without its limitations. Chemical inhibitors were used to disrupt Akt and Smad3 signaling. Although western blotting showed activated Akt was lowered to almost undetectable levels by MK-2206, and the overall ratio of activated Smad3 to total Smad2/3 content decreased in SIS3-treated groups, it is possible that some activity persisted, particularly for Smad3. Future studies in animal knockouts can control for small levels of activation in proteins of interest, though other off-target effects of the deletions may confound results. For example, a mouse knockout model of almost all Akt isoforms (*Akt1* ^+^/^−^, *Akt2* ^−^/^−^, and *Akt3* ^−^/^−^) is viable, but the animals display generalized abnormalities in metabolism and body weight [57]. Further cellular studies in 3-dimenstional (D) constructs using knockouts are also warranted, as previous in vitro models of tenogenesis have established the need for 3D culture in evaluating long-term tenogenesis [58]. 3D studies incorporating additional tenogenic markers, such as production of collagen I and III and proteoglycans, and evaluation of mechanical properties, are needed. Additionally, not all proteins of interest were assessed for 7 days. Proteins activated earlier in the pathway (for example, P-mTOR) were only quantified until 24 h or 3 d in either the MK-2206 + TGFβ2 or SIS3 + TGFβ2 experiments, though any notable changes in activation would likely occur within these time frames. Due to these short time frames associated with signaling activation (Akt activation was significantly increased at 30 min (Fig. 1b)), we assessed cell morphology and tenomodulin protein levels as markers of tenogenic induction. Our prior work showed scleraxis production in MSCs was not significantly increased until 14 and 21 d of TGFβ2 treatment [14]. Although some scleraxis production was detected at 7 d (data not shown) in the present study, the amount was not high enough to be quantified. Future studies using 3D constructs will need to assess gene expression and protein production of these tenogenic markers. We also only assessed Akt activation using a pan-Akt antibody, rather than examining individual Akt isoform activation. It is possible individual Akt isoforms impact tenogenic differentiation in different ways, though Akt isoforms are generally considered redundant, with overlapping functions in vivo [57]. Additionally, culture medium was supplemented with 50 ng/mL exogenous TGFβ2 every 3 days based on concentrations used in previous studies [11, 14, 59]. While this concentration is shown to be tenogenic in MSCs in vitro, it is possible it does not represent the in vivo availability and concentration of TGFβ2. We also did not supplement cells with other TGFβ isoforms, such as TGFβ1, or quantify TGFβ1 production by MSCs. Though TGFβ1 is generally considered fibrogenic [21, 22, 28] and its exogenous application is used in tendon injury models, it is possible that TGFβ1 activates alternative pathways when either Akt or Smad3 signaling is disrupted. As the goal of the present study was to assess a possible signaling mechanism by which TGFβ2 initiates early tenogenesis, supplementation with other TGFβ isoforms was outside the scope of the current investigation. Future studies will integrate assessment of alternative pathways, such as those associated with other tenogenic Smads (i.e., Smad4, Smad8), BMPs, and other TGFβ isoforms. Future studies in adult human MSCs using 3D culture systems will be needed to assess the potential benefits of clinical interventions targeting these pathways. Despite various limitations, this study represents a valuable contribution to the understanding of cell signaling pathways involved in the initial stages of TGFβ2-induced tenogenesis in MSCs and can be used to inform cellular studies of early tendon development.

## Conclusions

Overall, our findings show that Akt signaling is activated during TGFβ2-induced tenogenesis of MSCs, and Akt activation appears to impact tenogenic markers. Furthermore, disrupting Smad3 signaling and adding exogenous TGFβ2 did not prevent tenogenesis. Instead, tenogenic initiation appeared to be accelerated, with earlier increases in tenomodulin production and the appearance of tenogenic cell morphology. Collectively, our results suggest that Akt/mTORC1/P70S6K and other cell signaling pathways independent of Smad3 may be involved in tenogenesis. These pathways provide novel targets for future studies aiming to improve understanding of the cellular processes driving tenogenesis in stem cells.

## Supplementary Information

**Additional file 1: Figure S1.** mTOR activation is not impacted by TGFβ2 and is decreased with Akt inhibition. Quantified western blot band densitometry showing the ratio of P-mTOR to mTOR as a measure of mTOR activation levels in MSCs. Compared to vehicle controls, the ratio of P-mTOR to mTOR was not impacted significantly by TGFβ2 treatment at any timepoint. mTOR activation levels in MSCs were significantly decreased by MK-2206 compared to TGFβ2-only treated cells at (D) 24 h and (E) 3 d. * = *p* < 0.05. Bars = mean ± standard deviation.

**Additional file 2: Figure S2.** P70S6K activation increases with TGFβ2 and is unaffected by Akt inhibition Quantified western blot band densitometry showing the ratio of P-P70S6K to P70S6K as a measure of P70S6K activation levels in MSCs. (D) The ratio of P-P70S6K to P70S6K was significantly increased by TGFβ2 treatment at 24 h, and trended higher (C) at 60 m. P70S6K activation levels in MSCs were not significantly decreased by MK-2206 at any timepoint. * = *p* < 0.05. Bars = mean ± standard deviation.

**Additional file 3: Figure S3.** TGFβ2 activates Smad3 and SIS3 decreases the ratio of P-Smad3 to Smad2/3. Quantified western blot band densitometry showing the ratio of P-Smad3 to total Smad2/3 in MSCs treated with TGFβ2 and MK-2206 or SIS3. (A) The ratio of P-Smad3 to Smad2/3 increases significantly with MK-2206 + TGFβ2 treatment at 60 m. (B) Smad3 activation was comparable between all conditions at 24 h. (C) The ratio of P-Smad3 to Smad2/3 trends lower (*p* = 0.08, following 60 m of SIS3 treatment in MSCs. * = *p* < 0.05. Bars = mean ± standard deviation.

**Additional file 4: Figure S4.** mTOR activation is largely unaffected by SIS3. Quantified western blot band densitometry showing the ratio of P-mTOR to mTOR in MSCs treated with SIS3 to block Smad3 signaling. (A) The ratio of P-mTOR to mTOR was significantly decreased by SIS3 + TGFβ2 at 15 m, but mTOR activation was not significantly impacted at any other timepoint. * = p < 0.05. Bars = mean ± standard deviation.

**Additional file 5: Figure S5.** P70S6K activation is largely unaffected by SIS3. Quantified western blot band densitometry showing the ratio of P-P70S6K to P70S6K in MSCs treated with SIS3 to block Smad3 signaling. (A) The ratio of P-P70S6K to P70S6K was significantly decreased by SIS3, with and without TGFβ2, at 15 m. P70S6K activation was not significantly impacted by Smad3 inhibition at any other timepoint. * = p < 0.05. Bars = mean ± standard deviation.

**Additional file 6: Figure S6.** Akt activation as a function of tenogenic induction. Trend of the ratio of P-Akt to Akt indicates Akt activation in response to TGFβ2 peaks following 24 h of treatment. Akt activation then follows a decreasing trend, and is significantly lower compared to all other timepoints at 14 d. * = p < 0.05. Bars = mean ± standard deviation.

## Data Availability

All data and materials are available upon request.

## Abbreviations

DAPI: 4,6-Diamidino-2-phenylindole
ANOVA: Analysis of variance
BMP: Bone morphogenic protein
d: Days
DMSO: Dimethyl sulfoxide
DMEM: Dulbecco’s Modified Eagle’s Medium
E: Embryonic day
FBS: Fetal bovine serum
Hh: Hedgehog
mTOR: Mammalian target of rapamycin
MSCs: Mesenchymal stem cells
MAPK: Mitogen-activated protein kinase
P: Phospho-
PBS: Phosphate-buffered saline
PI3K: Phosphatidylinositol 3 kinase
P: Postnatal day
P70S6K: Protein S6 kinase
Smad: Small mothers against decapentaplegic
SDS: Sodium dodecyl sulfate
Tnmd: Tenomodulin
TGFβ: Transforming growth factor beta
TBST: Tris-buffered saline with 0.1% Tween20
VC: Vehicle Control
WB: Western blot
Wnt: Wingless/Integrated
