# Supplementary figures and images for: Akt signaling is activated by TGFβ2 and impacts tenogenic induction of mesenchymal stem cells
Source: Stem Cell Res Ther. 2021 Jan 26;12:88. doi: 10.1186/s13287-021-02167-2 (PMC7836508; doi:10.1186/s13287-021-02167-2)

# Phospho mTOR/mTOR

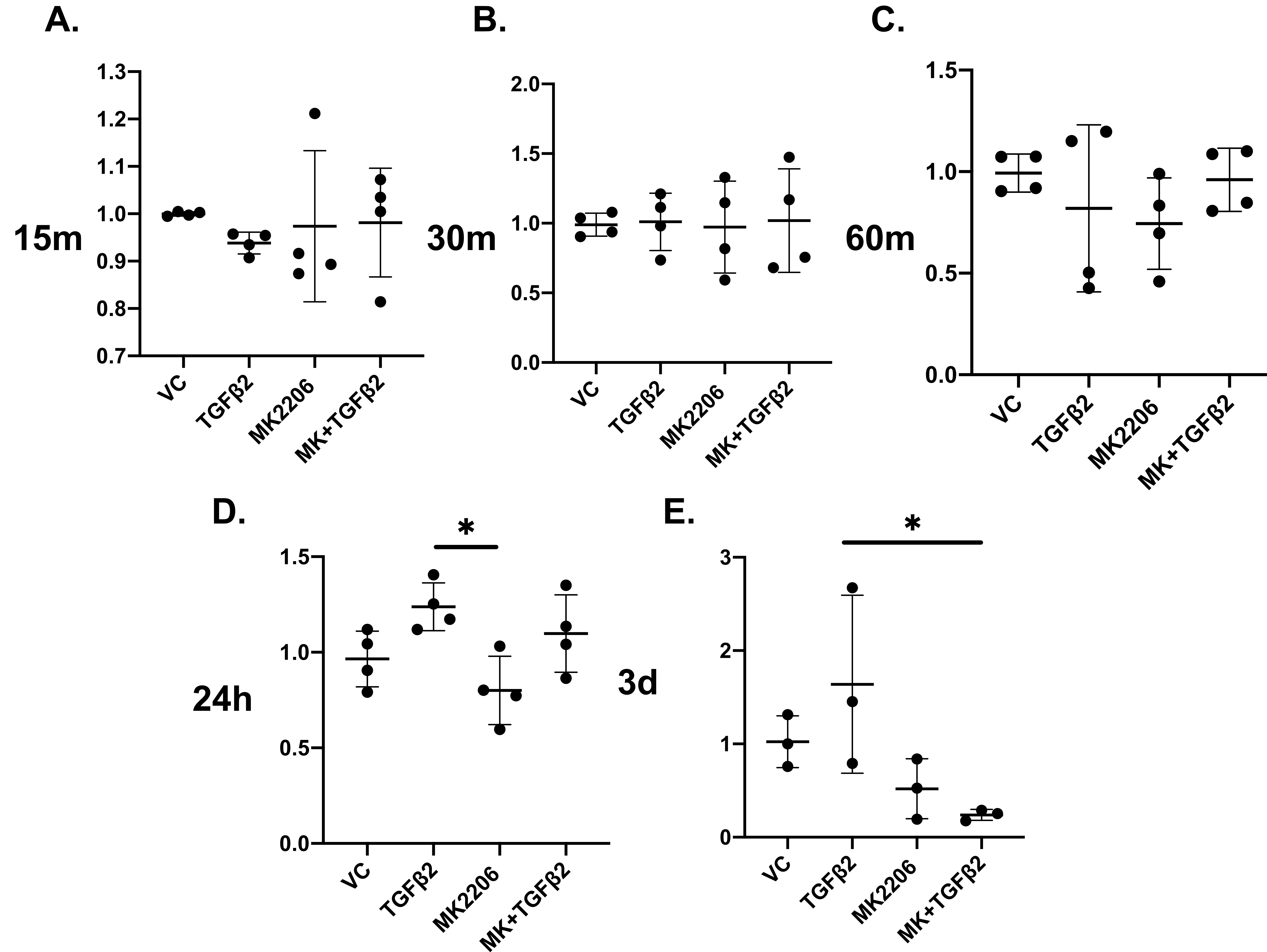

Supplement: Supplementary file 1 — Additional file 1: Figure S1. mTOR activation is not impacted by TGFβ2 and is decreased with Akt inhibition. Quantified western blot band densitometry showing the ratio of P-mTOR to mTOR as a measure of mTOR activation levels in MSCs. Compared to vehicle controls, the ratio of P-mTOR to mTOR was not impacted significantly by TGFβ2 treatment at any timepoint. mTOR activation levels in MSCs were significantly decreased by MK-2206 compared to TGFβ2-only treated cells at (D) 24 h and (E) 3 d. * = p < 0.05. Bars = mean ± standard deviation. [file 13287_2021_2167_MOESM1_ESM.pdf]

Phospho-P70S6K/P70S6K

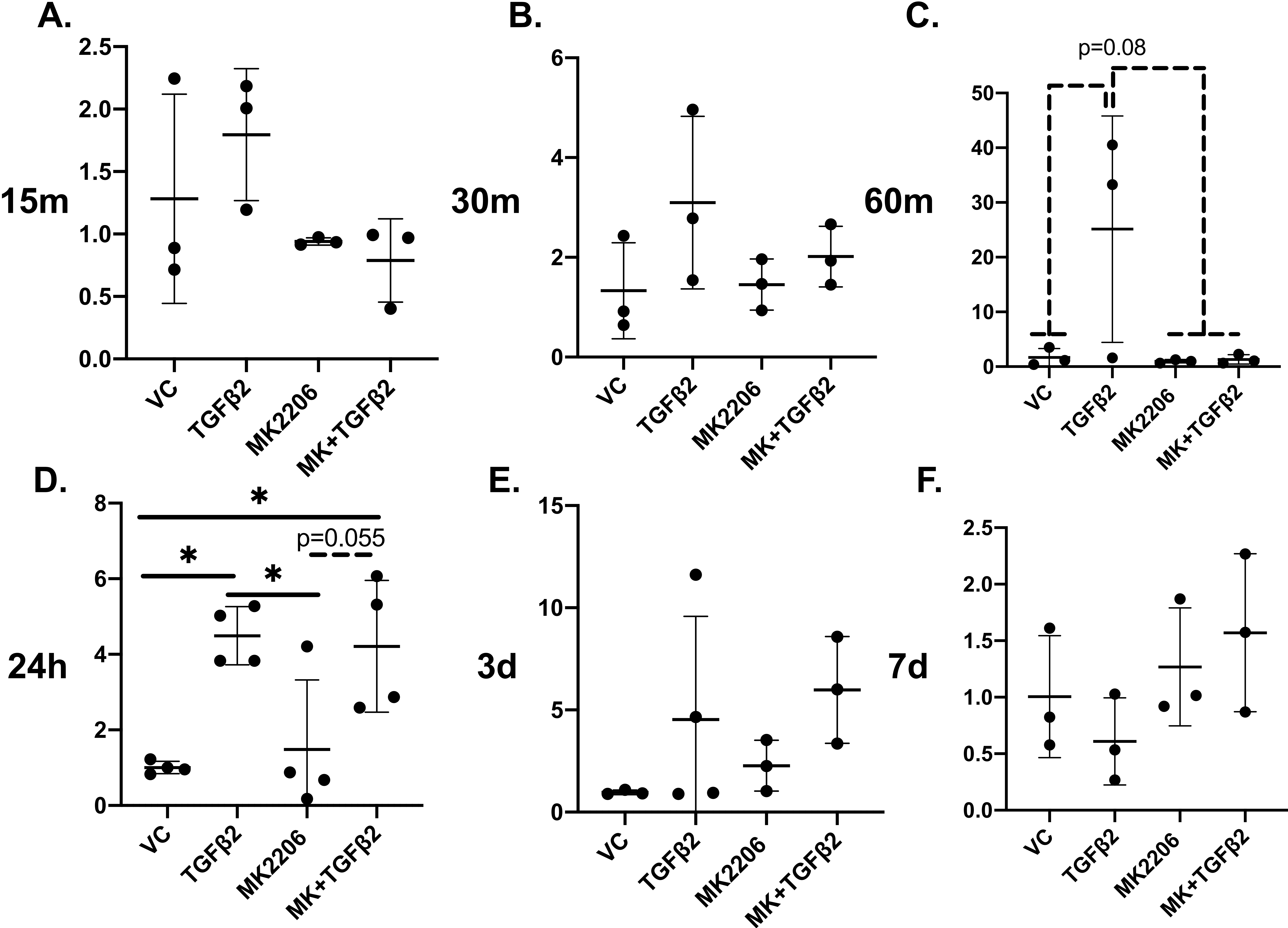

Supplement: Supplementary file 2 — Additional file 2: Figure S2. P70S6K activation increases with TGFβ2 and is unaffected by Akt inhibition Quantified western blot band densitometry showing the ratio of P-P70S6K to P70S6K as a measure of P70S6K activation levels in MSCs. (D) The ratio of P-P70S6K to P70S6K was significantly increased by TGFβ2 treatment at 24 h, and trended higher (C) at 60 m. P70S6K activation levels in MSCs were not significantly decreased by MK-2206 at any timepoint. * = p < 0.05. Bars = mean ± standard deviation. [file 13287_2021_2167_MOESM2_ESM.pdf]

# Phospho-Smad3/Smad3

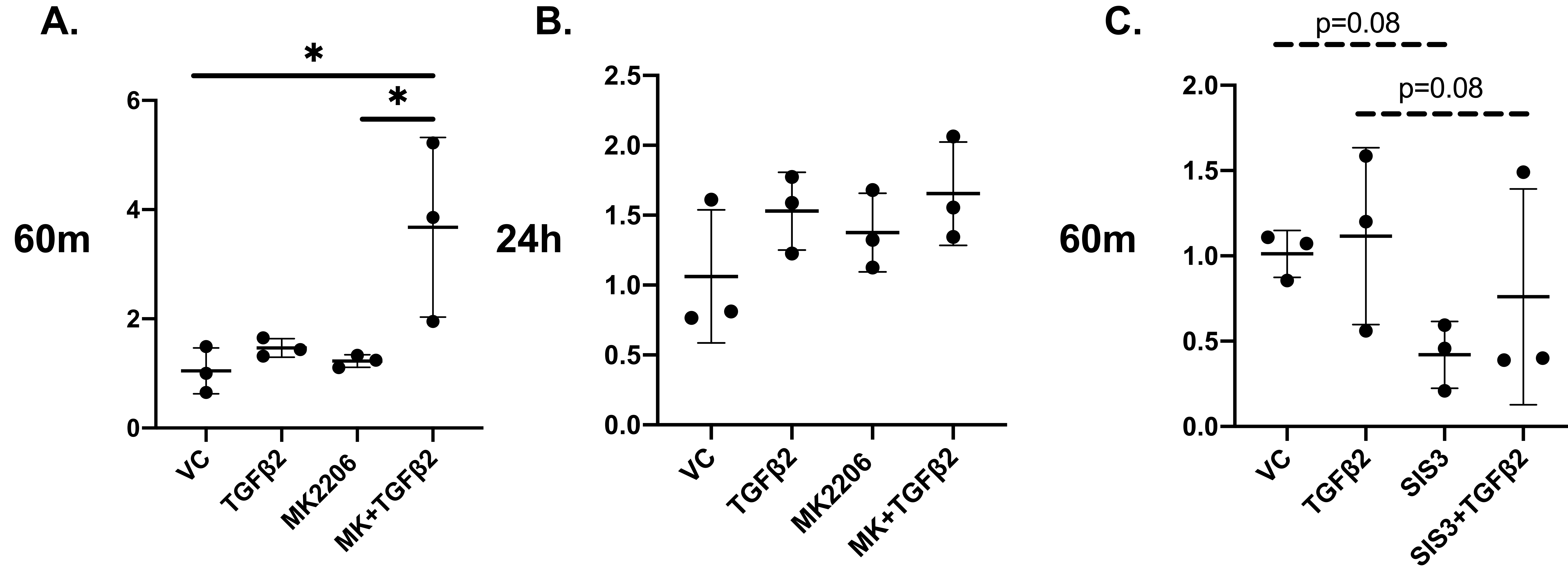

Supplement: Supplementary file 3 — Additional file 3: Figure S3. TGFβ2 activates Smad3 and SIS3 decreases the ratio of P-Smad3 to Smad2/3. Quantified western blot band densitometry showing the ratio of P-Smad3 to total Smad2/3 in MSCs treated with TGFβ2 and MK-2206 or SIS3. (A) The ratio of P-Smad3 to Smad2/3 increases significantly with MK-2206 + TGFβ2 treatment at 60 m. (B) Smad3 activation was comparable between all conditions at 24 h. (C) The ratio of P-Smad3 to Smad2/3 trends lower (p = 0.08, following 60 m of SIS3 treatment in MSCs. * = p < 0.05. Bars = mean ± standard deviation. [file 13287_2021_2167_MOESM3_ESM.pdf]

# Phospho-mTOR/mTOR

A.

15m

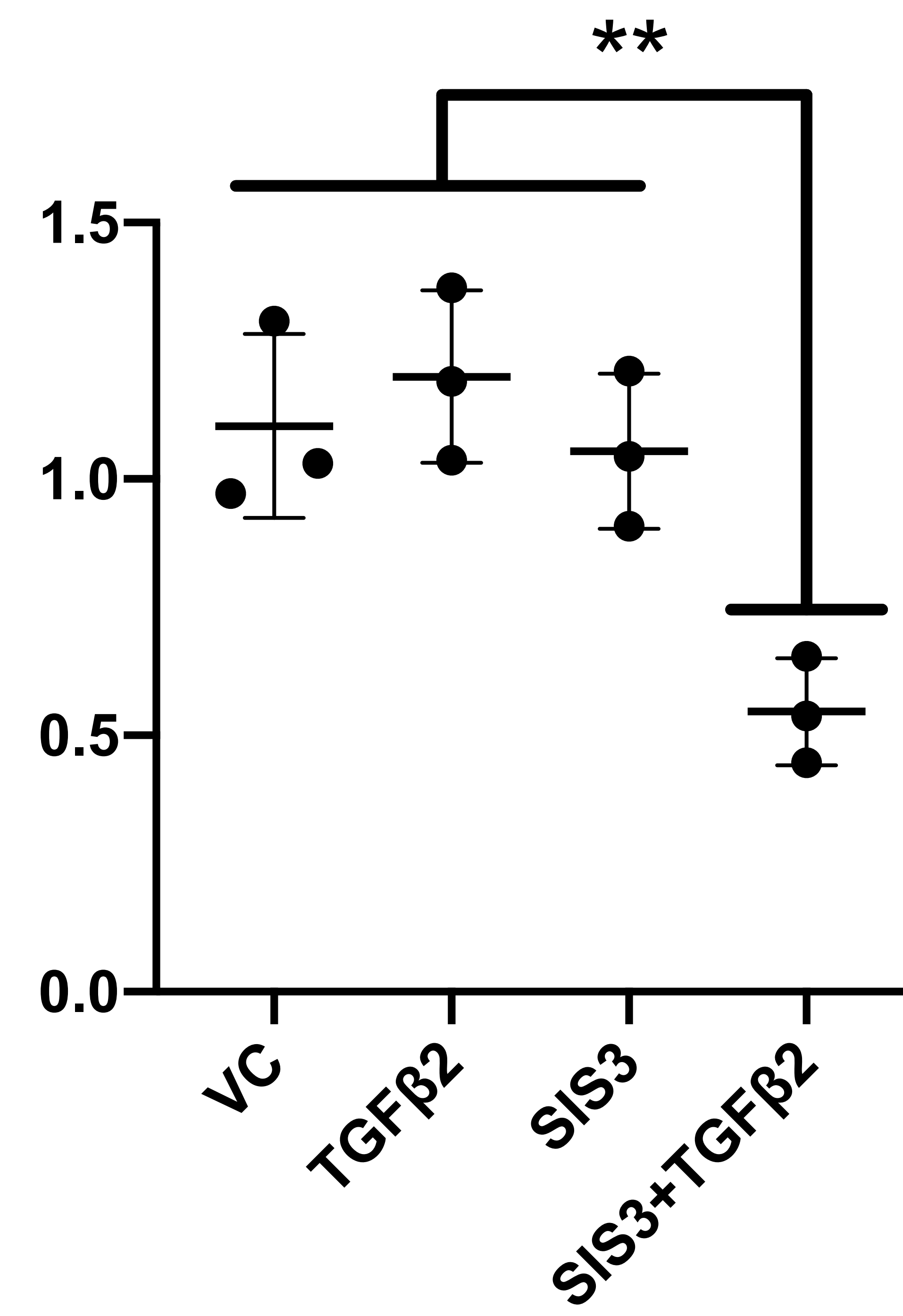

B.

30m

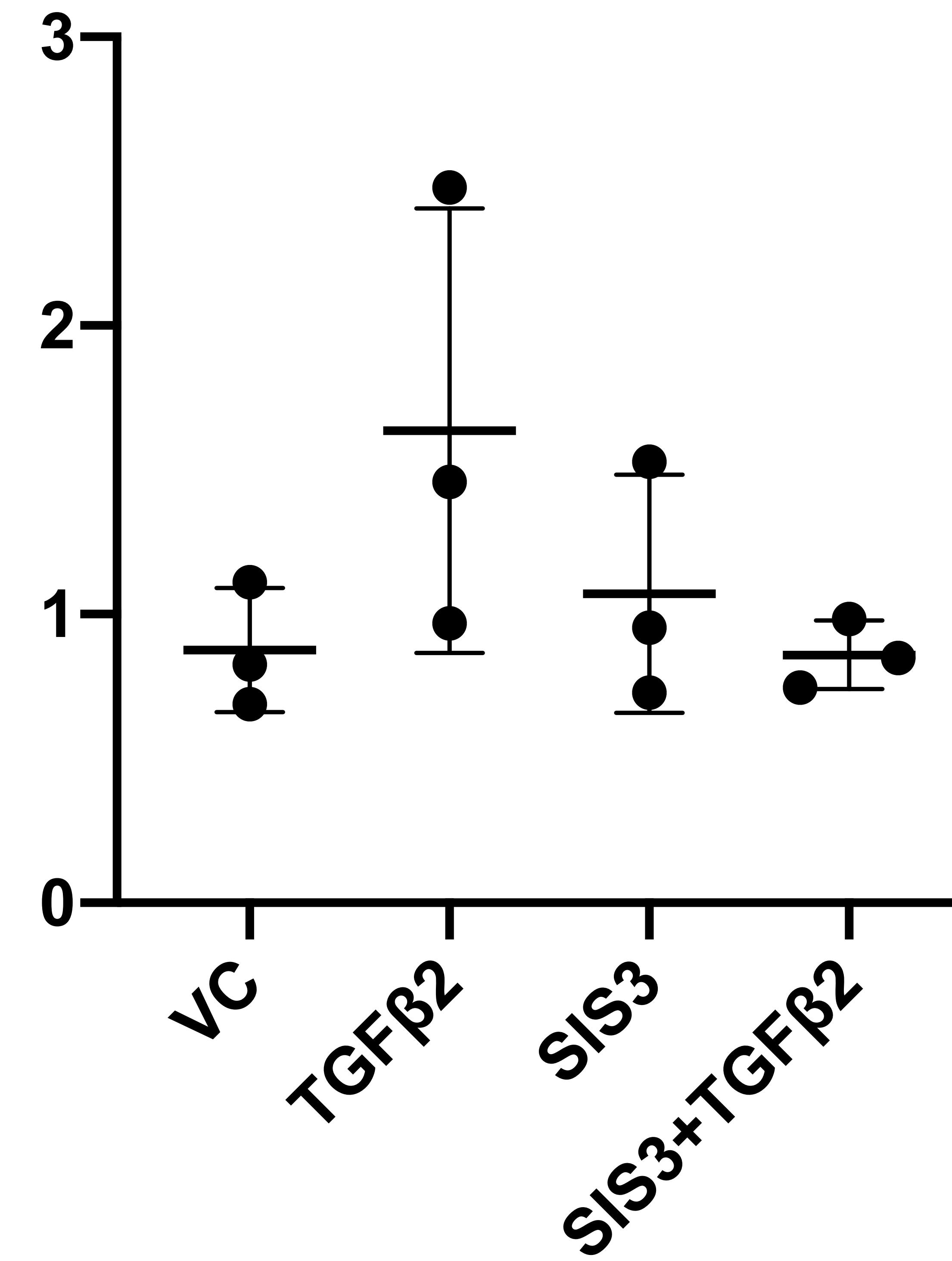

C.

60m

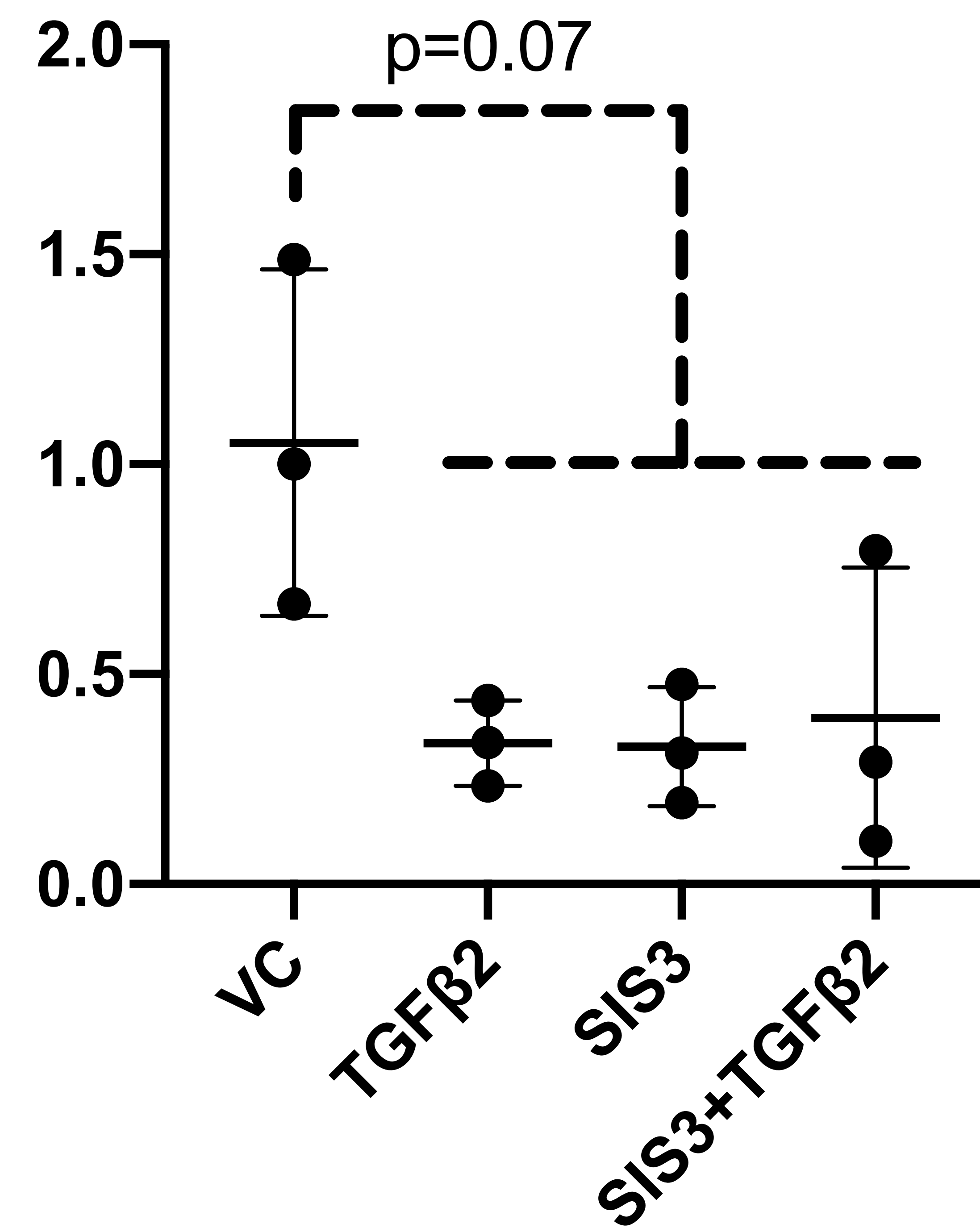

D.

24h

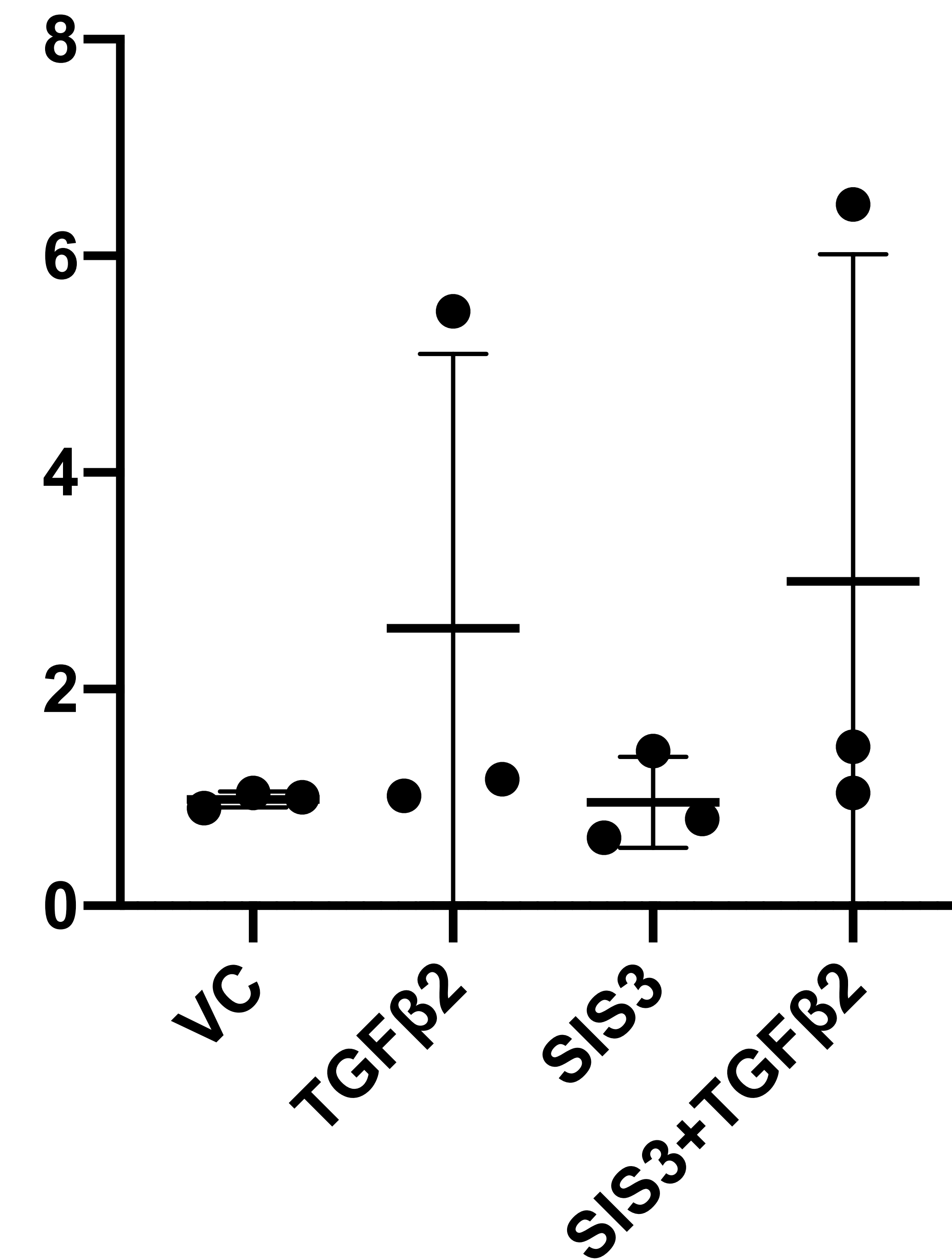

Supplement: Supplementary file 4 — Additional file 4: Figure S4. mTOR activation is largely unaffected by SIS3. Quantified western blot band densitometry showing the ratio of P-mTOR to mTOR in MSCs treated with SIS3 to block Smad3 signaling. (A) The ratio of P-mTOR to mTOR was significantly decreased by SIS3 + TGFβ2 at 15 m, but mTOR activation was not significantly impacted at any other timepoint. * = p < 0.05. Bars = mean ± standard deviation. [file 13287_2021_2167_MOESM4_ESM.pdf]

Phospho-P70S6K/P70S6K

A.

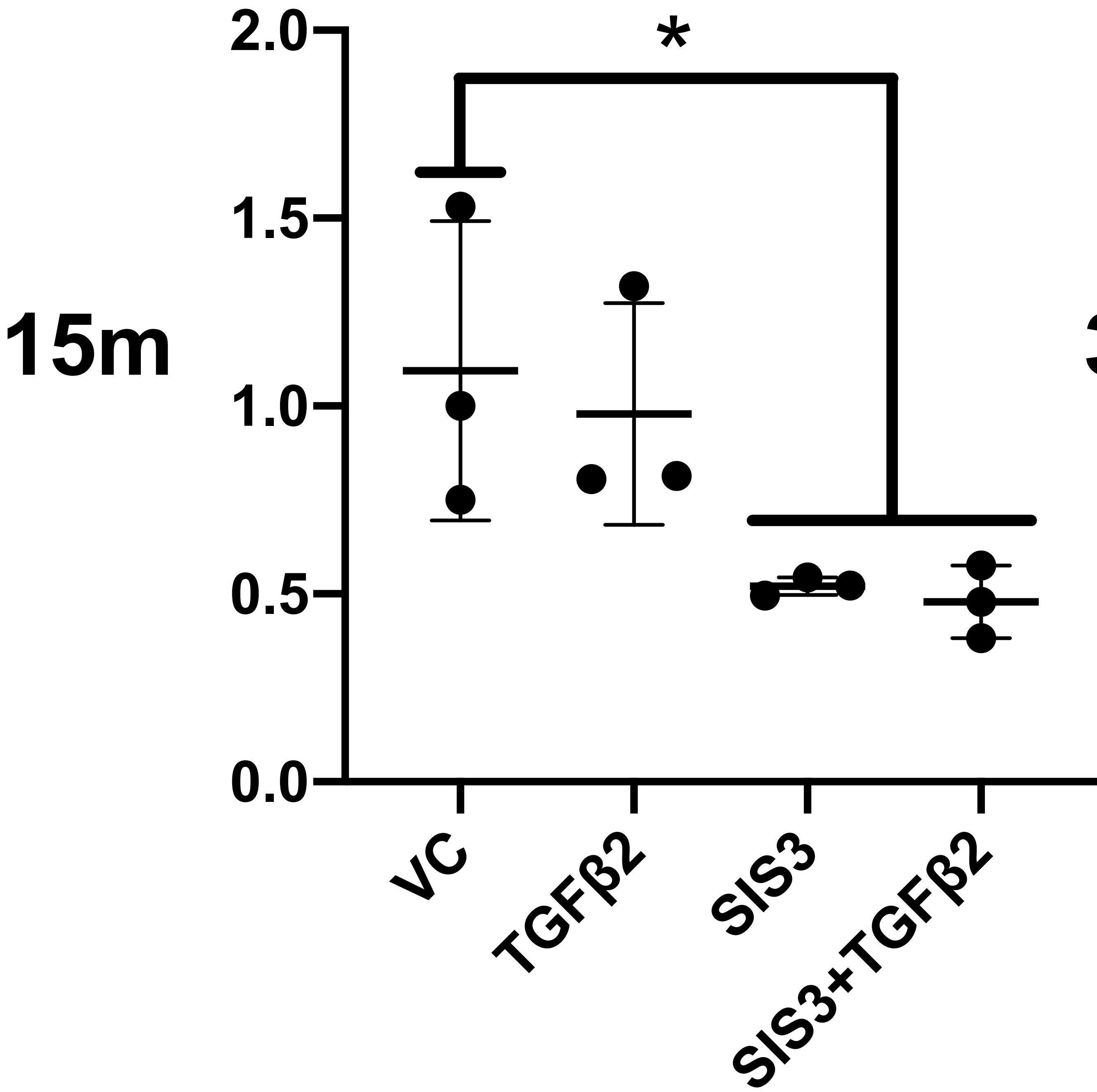

B.

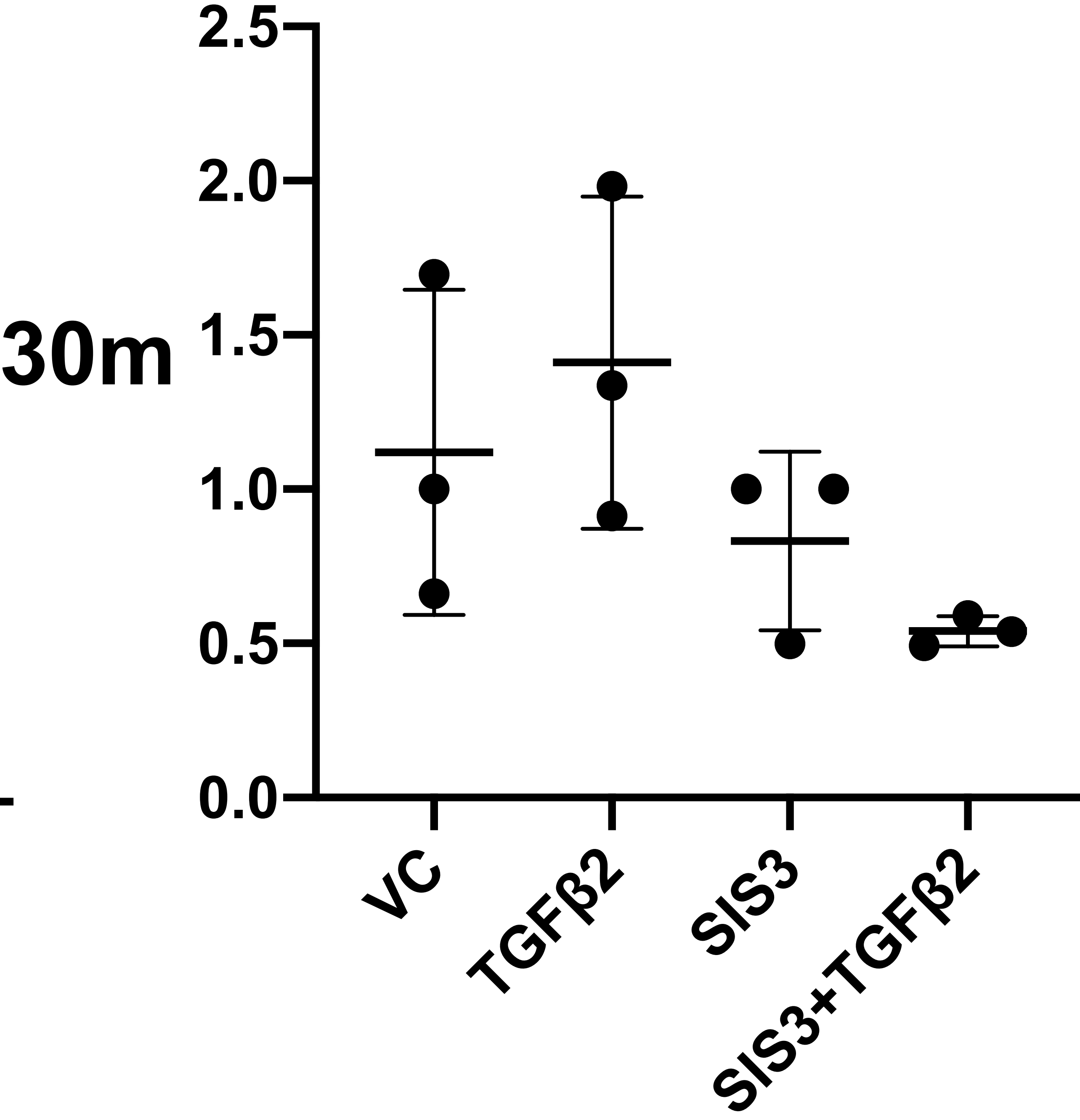

C.

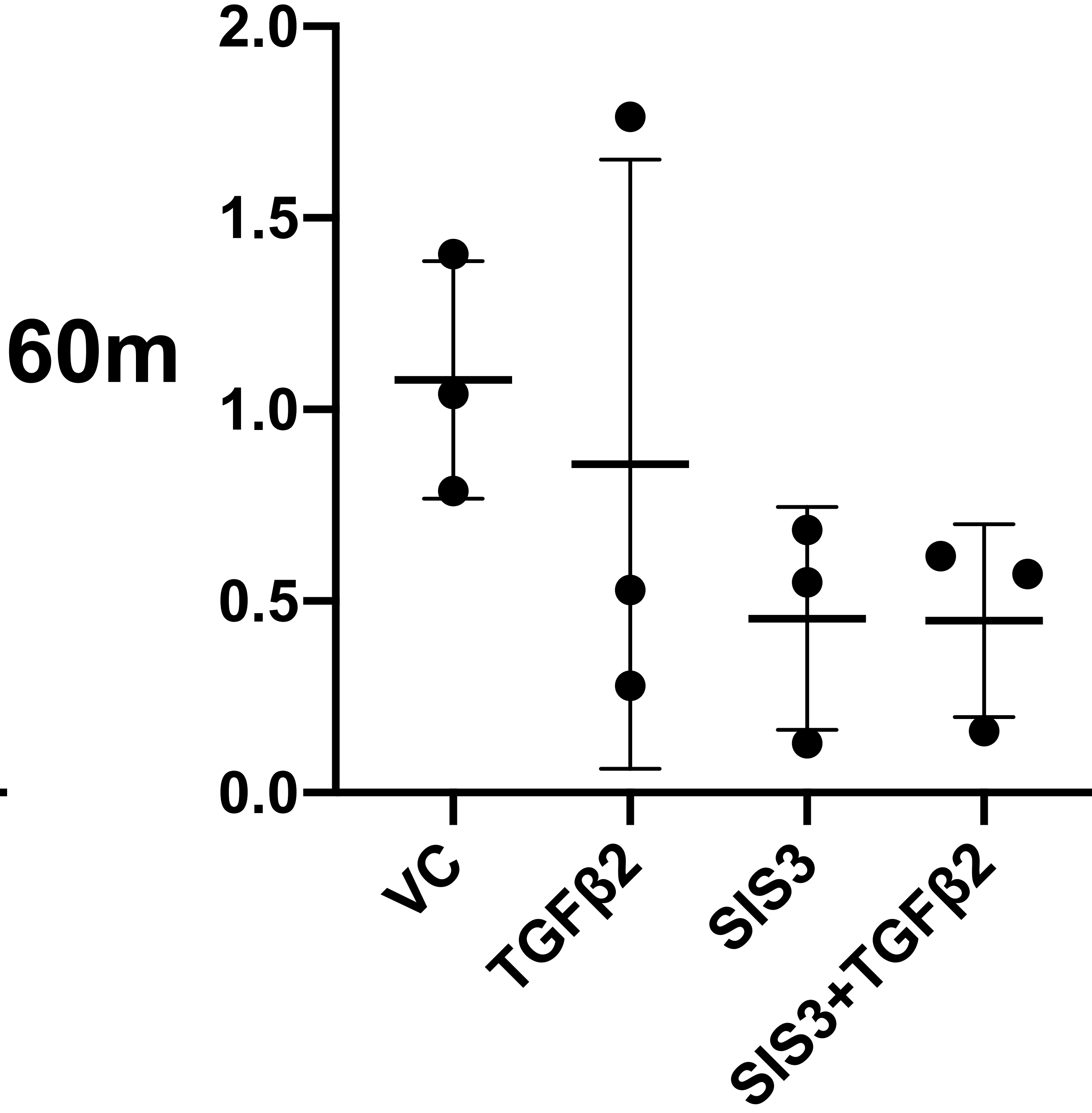

D.

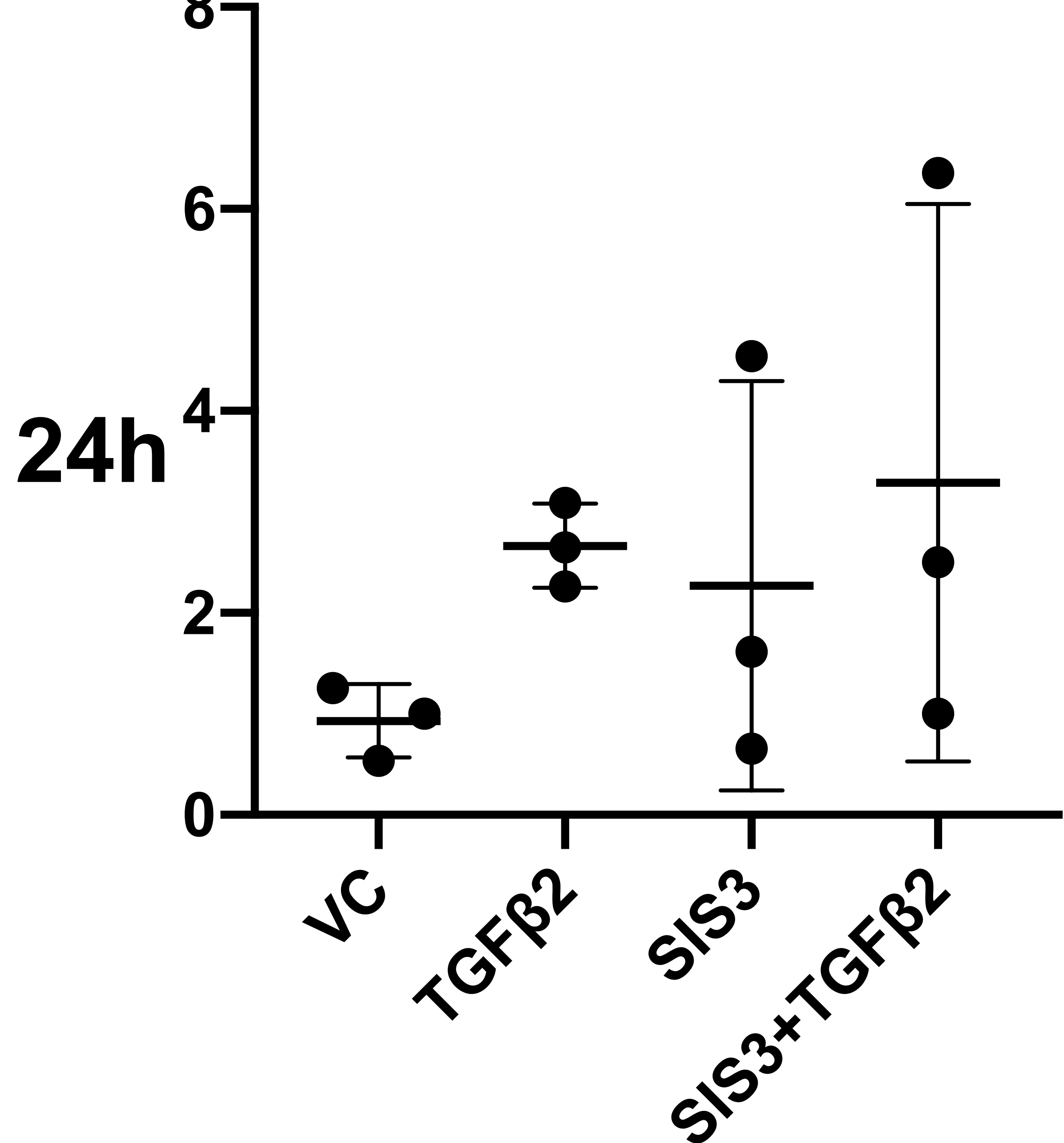

E.

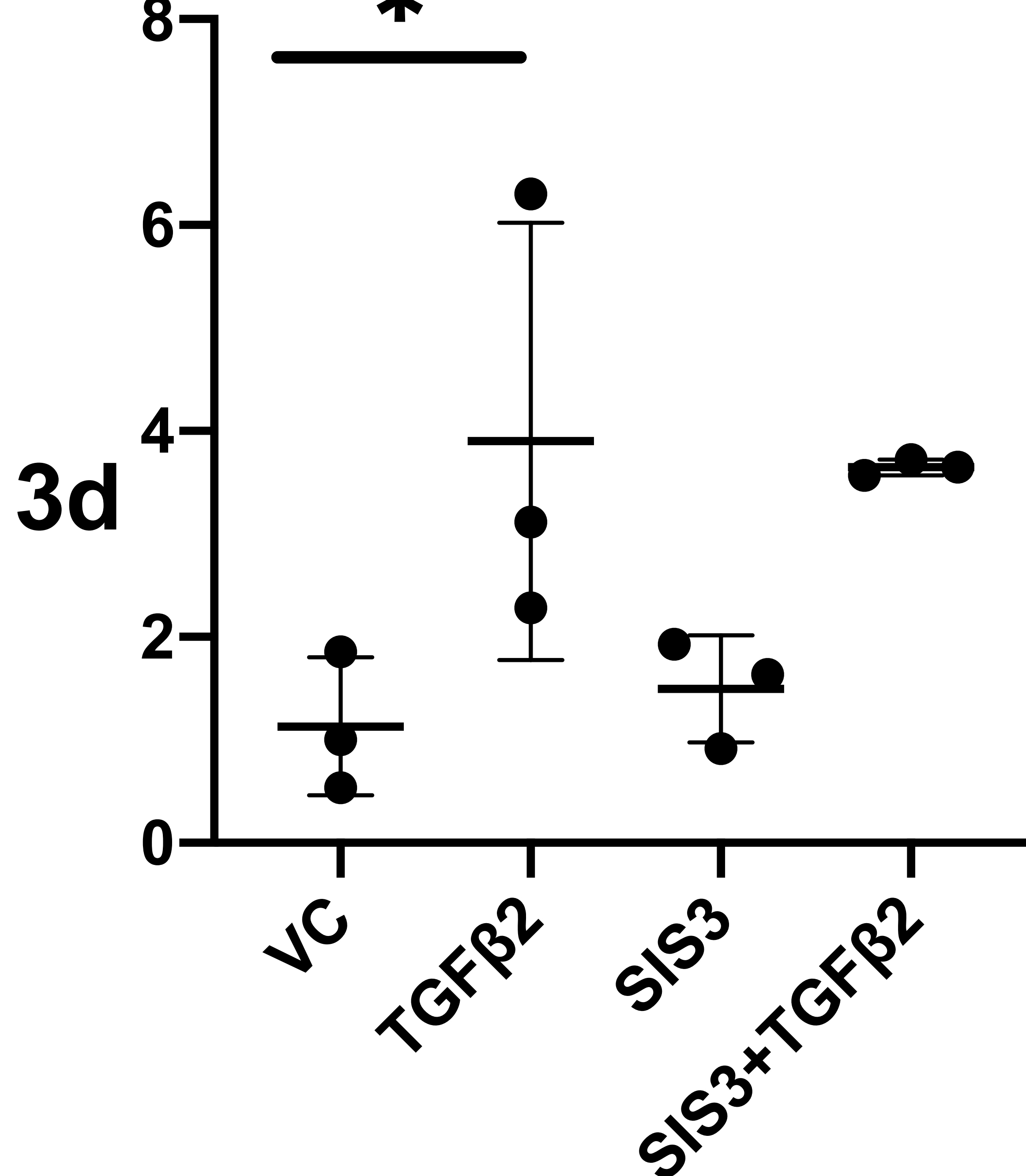

Supplement: Supplementary file 5 — Additional file 5: Figure S5. P70S6K activation is largely unaffected by SIS3. Quantified western blot band densitometry showing the ratio of P-P70S6K to P70S6K in MSCs treated with SIS3 to block Smad3 signaling. (A) The ratio of P-P70S6K to P70S6K was significantly decreased by SIS3, with and without TGFβ2, at 15 m. P70S6K activation was not significantly impacted by Smad3 inhibition at any other timepoint. * = p < 0.05. Bars = mean ± standard deviation. [file 13287_2021_2167_MOESM5_ESM.pdf]

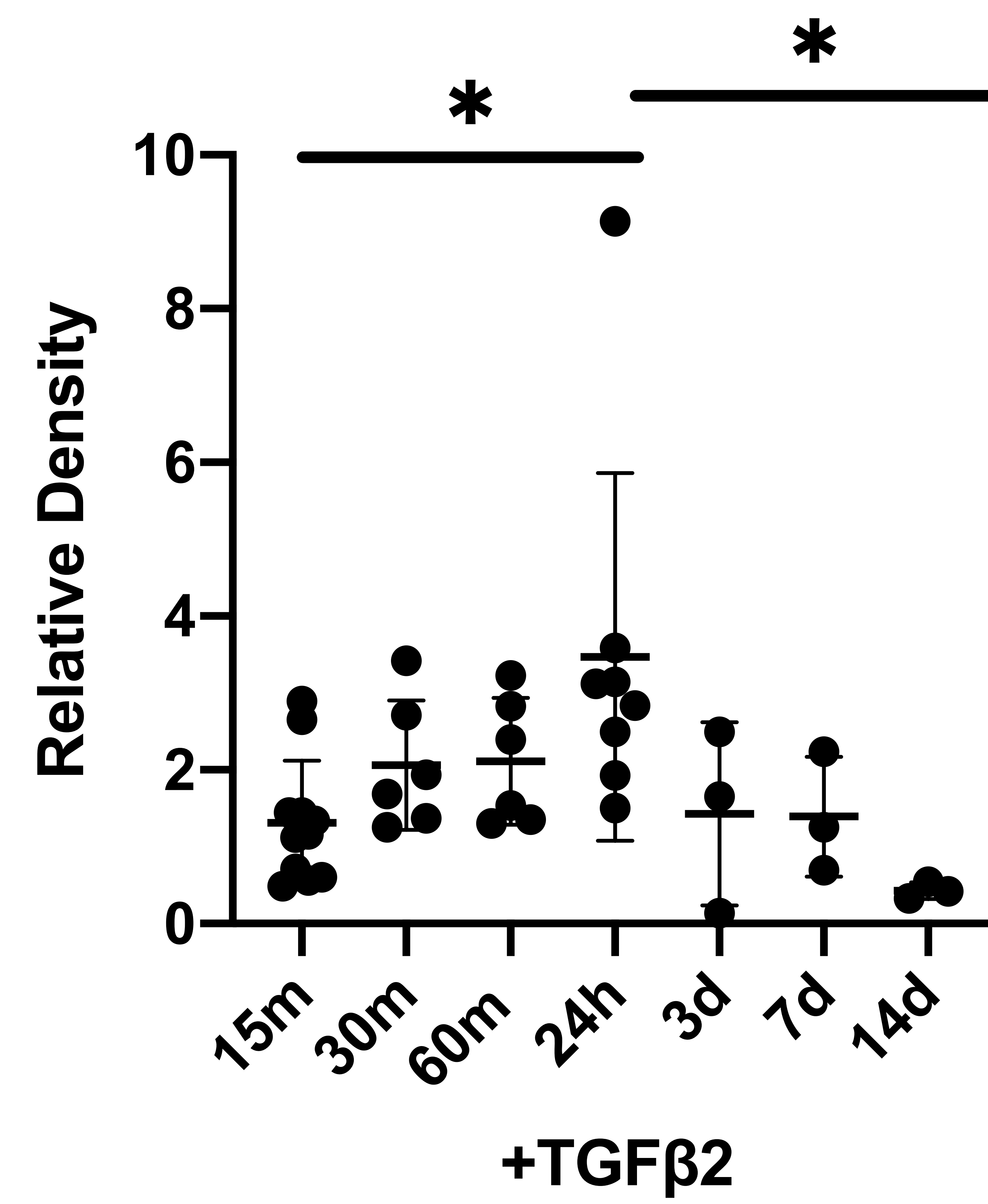

Supplement: Supplementary file 6 — Additional file 6: Figure S6. Akt activation as a function of tenogenic induction. Trend of the ratio of P-Akt to Akt indicates Akt activation in response to TGFβ2 peaks following 24 h of treatment. Akt activation then follows a decreasing trend, and is significantly lower compared to all other timepoints at 14 d. * = p < 0.05. Bars = mean ± standard deviation. [file 13287_2021_2167_MOESM6_ESM.pdf]
